# Highly Sensitive Detection of Anti-SARS-CoV-2 Antibodies in Human Serum Using Bloch Surface Wave Biosensor

**DOI:** 10.3390/s26010046

**Published:** 2025-12-20

**Authors:** Anastasiia Gaganina, Agostino Occhicone, Daniele Chiappetta, Paola Di Matteo, Norbert Danz, Matteo Allegretti, Peter Munzert, Chiara Mandoj, Francesco Michelotti, Alberto Sinibaldi

**Affiliations:** 1 Department of Basic and Applied Science for Engineering, Sapienza University of Rome, Via A. Scarpa 16, 00161 Rome, Italy; 2 Italian Institute of Technology (IIT), Center for Life Nano and Neuro Science, Viale Regina Elena 291, 00161 Rome, Italy; 3 Fraunhofer Institute for Applied Optics and Precision Engineering IOF, Albert-Einstein-Str. 7, 07745 Jena, Germany; 4 Translational Oncology Research Unit, IRCCS Regina Elena National Cancer Institute, Via Fermo Ognibene 23, 00144 Rome, Italy; 5 Clinical Pathology Unit and Cancer Biobank, IRCCS Regina Elena National Cancer Institute, Via E. Chianesi 53, 00144 Rome, Italy

**Keywords:** biosensors, one-dimensional photonic crystals, Bloch surface waves, fluorescence, SARS-CoV-2

## Abstract

Accurate and sensitive antibody detection remains critical for advanced COVID-19 diagnostics and monitoring SARS-CoV-2 immunity. This study presents a highly sensitive technique for detecting anti-SARS-CoV-2 antibodies in human serum using an integrated photonic sensing platform. The platform utilizes disposable one-dimensional photonic crystal biochips engineered to sustain Bloch Surface Waves. The biochips are integrated into a custom-made optical set-up, which is capable of dual-mode detection: label-free refractometry and label-based fluorescence. Tests on human serum, including negative controls and positive samples from a recovered COVID-19 patient, confirmed the platform’s effective performance. In fluorescence mode, clear discrimination between positive and negative samples was achieved down to a 1:10^4^ serum dilution, with an optimal operating range centered around 1:10^3^ dilution. These results demonstrate the potential of the technique as a highly sensitive and versatile platform for antibody detection, with significant relevance for advanced COVID-19 diagnostics.

## 1. Introduction

The global outbreak of Coronavirus Disease 2019 (COVID-19) [1], caused by Severe Acute Respiratory Syndrome Coronavirus 2 (SARS-CoV-2), posed a global health crisis [2,3]. The urgent need for rapid and accurate testing technologies drove intense research. COVID-19 thus served as a catalyst for the advancement of next-generation biosensing platforms.

The widely accepted gold standard for detecting active infection is Reverse Transcription–Polymerase Chain Reaction (RT-PCR), which identifies the virus’s genetic material with exceptional sensitivity and specificity [4]. However, RT-PCR is limited by its high cost, long turnaround times, and dependence on centralized laboratory infrastructure, making it unsuitable for rapid, high-throughput point-of-care (POC) applications and large-scale serological surveillance [5,6]. In recent years, POC systems have gained widespread use compared to conventional laboratory methods, enabling faster diagnostic results by allowing the detection of pathogenicity at the molecular level and identifying a wide range of disease agents [7,8]. Consequently, POC systems play a vital role in providing quick and accurate test results across various clinical settings [7]. These systems are especially valuable in emergency departments, where they contribute to improved patient care and infection control [9]. Moreover, the expansion of POC technologies has significantly advanced the reach of personalized medicine [7].

At the same time, Enzyme-Linked Immunosorbent Assays (ELISA) remain the established standard for antibody quantification due to their reliability and cost-effectiveness [10,11,12]. Nevertheless, traditional ELISA formats are time-consuming, labor-intensive, and limited in sensitivity and multiplexing capability, thus reducing their applicability in high-throughput applications [11,12,13]. Achieving ultrasensitive detection in complex matrices, such as those in the sub-pg/mL range, often necessitates additional signal amplification and assay modification steps that increase complexity and duration [13]. Similarly, Lateral Flow Assays (LFAs), while providing rapid and decentralized testing, generally exhibit limited sensitivity, meaning that the organism’s antibody production may not be detectable during the early phase of infection [12,14]. Consequently, negative LFA results cannot reliably exclude COVID-19 infection or disease. These tests should therefore not replace molecular diagnostics in acute-care settings but may serve as complementary triage tools when acknowledging their limitations and anticipating their logistical benefits [12,15]. Recently, POC nanobiosensors have emerged as one of the most promising diagnostic tools, leveraging nanomaterials to achieve more efficient and rapid detection outcomes [16].

In this work, we present human serum analysis performed with an integrated optical sensing platform. This platform utilizes disposable biochips with one-dimensional photonic crystals (1DPCs) engineered to support Bloch Surface Waves (BSWs) [17]. The strength of our system lies in dual-mode detection on a single disposable biochip: label-free refractometry to monitor binding kinetics and label-based fluorescence with a high signal-to-noise ratio.

BSW biosensors are emerging as a valuable alternative to traditional immunoassay platforms such as ELISA and CLIA [18]. Instead of depending on enzymatic labels and complex, time-consuming procedures, BSW systems support real-time, label-free biomolecular detection with exceptional sensitivity. Their unique optical architecture, based on electromagnetic field confinement at a photonic crystal interface, significantly boosts interactions with captured analytes, enabling lower detection limits and faster analytical responses. In addition, the photonic crystal structure of BSW devices can be engineered to tailor their optical behavior, facilitating multiplexed measurements and compatibility with a wide range of diagnostic targets. By merging the robustness of established biochemical assays with the benefits of optical sensing, such as reduced sample handling, quick data acquisition, and straightforward miniaturization, BSW biosensors pave the way for compact and efficient point-of-care diagnostic solutions.

In previous studies, some of the authors have demonstrated the applicability of the proposed technique for detecting inflammatory and cancer biomarkers [19,20], miRNA [21], as well as for investigating the diffusion coefficients of various aqueous solutions [22]. In particular, they provided a proof of concept for the detection of anti-SARS-CoV-2 antibodies in human serum, thereby validating the potential of the approach for infectious disease diagnostics [23]. In the present work, fluorescence functionality was achieved using nanocrystals (quantum dots, QDs) as fluorescent labels, characterized by their narrow emission spectra [24]. Therefore, owing to their intrinsic excitation properties, QDs effectively minimize the reabsorption phenomena compared to those previously reported for organic dyes [24,25].

Although focused on detection of anti-SARS-CoV-2 antibodies, this study primarily demonstrates the high sensitivity of the platform. Its robust performance and dual-mode capability make it a promising and versatile tool for advanced diagnostics, enabling sensitive detection of antibodies and biomarkers across a wide range of diseases.

## 2. Materials and Methods

### 2.1. Read-Out Platform

**Figure 1:**
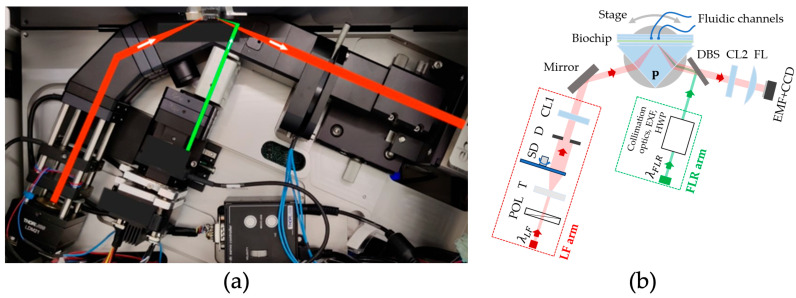
(**a**) Photo of the optical platform: with red is shown the LF path, and with green the FLR path. (**b**) Schematic representation of the platform. Coupling prism (P), Polarizer (POL), Cylindrical lens (CL), Fourier Lens (FL), telescope (T), rotating scattering disk (SD), diaphragm (D), dichroic beam splitter (DBS). EXF, EMF and HWF are excitation filter, emission filter and half-wave plate, respectively.

The read-out apparatus is shown in Figure 1a and can be operated in either label-free (LF) or label-based fluorescence (FLR) mode [21,23].

The LF sensing mechanism relies on the excitation of BSWs featuring an evanescent electromagnetic field highly sensitive to dielectric variations occurring at the biochip’s surface [17,19,20,22,23,26]. Therefore, when target molecules bind to surface-bound receptors, they cause an alteration in the local refractive index [27], paving the way to real-time monitoring change in the effective refractive index at the biochip’s surface. This change is detected as an angular shift in the BSW resonance angle, providing quantitative information on molecular binding kinetics [20,23]. With reference to the setup sketch of Figure 1b, the LF arm is illuminated by a laser diode with wavelength λ_LF_ = 671 nm; that is, TE polarized with a polarizer (POL) and expanded by a telescope (T). A rotating scattering disk (SD) destroys spatial coherence. After that, the central portion of the beam is selected with a circular diaphragm (D) and is focused with a cylindrical lens (CL1) onto the coupling prism.

In the FLR mode, the system simultaneously excites the fluorophore labels exploiting the BSW resonance as well. This results in a highly amplified, angularly resolved fluorescence signal, coupled to the evanescent electromagnetic field of the BSW [21,23,28]. Referring again to Figure 1b, in FLR arm, a laser diode emitting at wavelength λ_FLR_ = 637 nm is collimated and focused with a cylindrical lens to a strip on the chip surface. The collimation optics, the excitation filter (EXF) and the half-wave plate (HWP) used to set the polarization to TE are also arranged inside the arm. A dichroic beam splitter (DBS) reflects the original beam and transmits fluorescence emission from the biochip surface.

The total internal reflection condition required for BSW excitation is achieved via a coupling prism (P in Figure 1b) achieving a Kretschmann–Raether configuration. The buffer and the sample solutions are injected into the microfluidic channel by means of a syringe pump (4-port Cavro^®^ Centris, Tecan Trading AG, Männedorf, Switzerland).

The design of the optical detection system ensures that the same angular range is observed for all the spots along the illuminated region on the biochip surface for both LF (2.7 deg) and FLR (8.0 deg) modes. The reflected beam is imaged by a cylindrical lens (CL2) and a cylindrical Fourier lens (FL) onto a monochrome CCD camera (Sony ICX814, resolution 3388 × 2712 pixels, Apogee Ascent, North Logan, UT, USA). An emission filter (EMF) in front of the CCD cuts stray light from the excitation FLR beam; such filter transmits at λ_LF_, therefore preserving the LF operation [23]. The enhanced emission is collected by a CCD camera over an angular range of 8.0 deg, corresponding to 3388 camera pixels. For spatial mapping, 2712 pixels are used and divided into 90 spots of 30 pixels each. In this way, a 2D FLR map is obtained, providing both angular and spatial information on the fluorescence intensity emitted from the biochip’s surface. The 2D FLR maps were acquired at several integration time settings of the camera, while all analyses were conducted under the same exposure setting (I = 10^−3^ s).

### 2.2. Biochips

**Figure 2:**
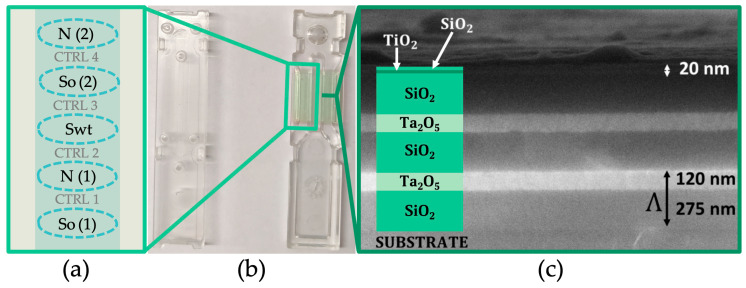
(**a**) Arrangement of the signal regions on the biochip surface. The regions were physically spotted according to the scheme shown, alternating N, So, and Swt proteins. (**b**) Photo of the two parts of the disposable biochip: the fluidic element (left) featuring microfluidic channels, and the sensor element (right) with the 1DPC multilayer. (**c**) FESEM image of the cross-section of the 1DPC.

Figure 2b shows a photograph of the biochip, which is composed of two components that can be mechanically coupled to form a sealed microfluidic cell, ensuring stable and leak-free operation during the assay. The biochip substrate is an optical quality plastic material made of a cyclic olefin copolymer (TOPAS 5013 LS, TOPAS Advanced Polymers GmbH, Raunheim, Germany) onto which the 1DPC multilayer is deposited by plasma ion-assisted evaporation.

As shown in the FESEM image in Figure 2c, the 1DPC is composed of two and a half periods of SiO_2_ and Ta_2_O_5_ with refractive indexes n_SiO2_ = 1.45 and n_Ta2O5_ = 2.09 at λ_LF_ and nominal thicknesses 275 nm and 120 nm, respectively. A thin double layer of TiO_2_ (n_TiO2_ = 2.28 at λ_LF_) and SiO_2_, both with nominal thicknesses equal to 20 nm, cover the periodic stack. The role of this top layer is to support the surface mode, to tune the losses of the 1DPC via TiO_2_ for optimal label-free operation, and to provide a SiO_2_ termination layer for the chemical surface functionalization with silanization methods [19,20,29].

### 2.3. Chemical Functionalization, Bio-Conjugation Procedure and Assay Format

The chemical functionalization process begins with cleaning the surface of the 1DPC biochips using a piranha solution (a 3:1 mixture of sulfuric acid and 30% hydrogen peroxide) for 10 min. The biochips are then thoroughly rinsed with deionized (DI) water and dried with a stream of air. This procedure effectively removes all organic contaminants and exposes hydroxyl groups for subsequent functionalization. Next, the plastic biochips are immersed in a 2% solution of (3-aminopropyl) triethoxysilane (APTES, Sigma-Aldrich, Merck, Darmstadt, Germany) in pure ethanol (99.8%) at ambient temperature for 1 h [29]. After removal from the APTES solution, the chips are sonicated, rinsed with ethanol, and soft-baked on a hot plate at 60 °C for 1 h. The APTES-modified chips are then reacted with a 1% (*v*/*v*) solution of glutaraldehyde (Sigma-Aldrich, Merck, Darmstadt, Germany) in 100 mM sodium bicarbonate buffer (pH 8.5) containing 0.1 mM sodium cyanoborohydride (Sigma-Aldrich, Merck, Darmstadt, Germany) for 1 h at ambient temperature. This is followed by another round of sonication and rinsing with DI water. At this stage, the glutaraldehyde-activated surface of the biochip is ready for the bio-conjugation process.

The bio-conjugation involves two main steps: probe molecule immobilization and surface blocking.

First, the probes are spotted onto the functionalized biochip surface in defined regions of interest (ROIs) using a Nano-Plotter (GeSiM, Radeberg, Germany). The GeSiM’s Nano-Tip parameters are configured as voltage (V = 68 V), pulse width (w = 100 μs), and frequency (f = 100 Hz). For each ROI, a line of six drops is spotted along a 1.25 mm segment perpendicular to the fluidic channel to promote the merging of drops. Each of these drops consists of 15 nano-droplets (all approximately 700 pL in volume). So, the total volume of the solution in the ROI is approximately 63 nL, and its resulting size is approximately 560 µm. The distance between the ROIs is about 470 µm. The system ensures an XY-axis positional accuracy of ±10 µm, making it a reliable tool for protein array preparation. The arrangement of the ROIs is illustrated in Figure 2a. The regions include: two ROIs of N protein (nucleocapsid protein, Sino Biological Europe GmbH, Eschborn, Germany), two ROIs of So (omicron spike variant, sub-variant BA.4/BA.5, Sino Biological Europe GmbH, Eschborn, Germany), and one of Swt (wild type spike, Life Technologies, Milano, Italy). After 1 h of incubation at 16 °C in a controlled humid environment (85%), the biochip is rinsed with PBS 1× and dried under an air stream. Subsequently, the biochip is coupled with its microfluidic counterpart and mounted on the platform (for more details see SM of [23]).

**Figure 3:**
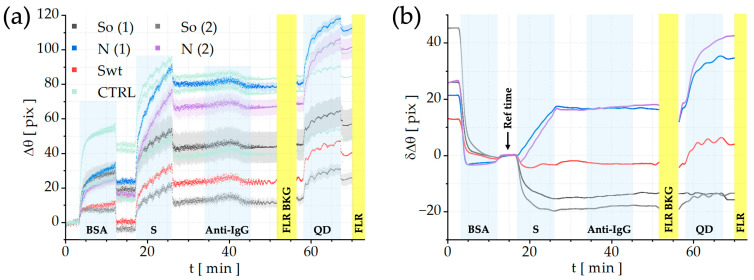
(**a**) LF sensogram obtained for the positive sample diluted 1:10^3^. (**b**) Differential LF sensogram obtained for the positive sample 1:10^3^ diluted for N (blue, purple curves), So (grey, black curves), and Swt (red curve) regions.

Second, for surface blocking, a solution of bovine serum albumin (BSA, Sigma-Aldrich, Merck, Darmstadt, Germany) is introduced into the microfluidic cell. Its role is to passivate residual binding sites and minimize non-specific adsorption on the surface (the first assay step, as shown in the sensograms of Figure 3a).

The assay shown in Figure 3a proceeds with the sequential injection of human serum samples (S step), followed by incubation with biotinylated anti-human IgG antibodies (Bio-Rad Laboratories, Hercules CA, USA, 1:20, Anti-IgG step). Finally, streptavidin-coated quantum dots (Streptavidinated QD655 from Life Technologies, Milano, Italy, emission peak at 655 nm) are introduced to confer fluorescence functionality to biochip (QD step). Fluorescence maps are acquired before and after injection of fluorescent labels to obtain background and signal data for accurate analysis.

The chosen configuration of surface functionalization (S and N proteins, accompanied by BSA control areas) enabled us to obtain LF and FLR responses that were largely unaffected by non-specific interactions or surface-related artifacts on the 1DPC platform.

All reagents were injected at a flow rate of 1.37 µL/s. A total volume of 180 µL was delivered for each injection, providing a contact time of 10 min with the sensitive area of the biochips for human serum, anti-IgG, and strept-QDs solutions, as shown in Figure 3.

### 2.4. Human Serum Samples

Blood samples were obtained from a representative cohort of healthy donors and neoplastic patients with a documented history of SARS-CoV-2 infection. Samples were sourced from the IRCCS Regina Elena National Cancer Institute (IRE) Biobank (Rome, Italy), following the signing of a dedicated written informed consent. Whole blood was processed within 1 h of collection, and sera were isolated by centrifugation at 2000× *g* for 20 min. Aliquots were stored at –80 °C in single-use tubes, each labeled with a unique, randomized barcode. The antibody concentration for each undiluted serum sample was quantified using two distinct chemiluminescence immunoassay (CLIA) platforms: the Maglumi 2019-nCoV IgG Immunoassay (Snibe Diagnostics, Shenzhen, China, threshold: 1 AU/mL) and the Liaison SARS-CoV-2 S1/S2 IgG assay (Diasorin S.p.a., Saluggia, Italy, threshold: 15 AU/mL). Two reference sera were selected for the subsequent assays: a negative serum (NEG), taken from a patient with no prior SARS-CoV-2 infection (Maglumi: 0.1 AU/mL), and a positive serum (POS), taken from a patient who had recovered from SARS-CoV-2 infection and had been tested negative by PCR in the preceding 86 days (Maglumi: 22.2 AU/mL; Liaison: 383 AU/mL). For the assays conducted with the 1DPC biochips, both the negative and positive serum samples were diluted in PBS 1X containing 0.1% BSA. The diluted samples were gently mixed and equilibrated to room temperature before injection into the microfluidic system.

## 3. Results and Discussion

The human samples analyzed in this work were obtained from a broader study cohort comprising blood samples from both healthy volunteers and cancer patients with a documented history of SARS-CoV-2 infection. To demonstrate the detection protocols developed here, only two samples (in different dilutions) from this cohort were analyzed. The findings from this preliminary work will guide a follow-up study using optimized 1DPC biochips, allowing the inclusion of a larger sample set and enabling robust statistical evaluation through Passing–Bablok regression and Bland–Altman analysis.

### 3.1. LF-Based Detection

The LF mode plays a pivotal dual role within our platform: it provides real-time monitoring of the entire assay process while simultaneously enabling quantitative characterization of each bio-conjugation step. Since the effective refractive index on the biochip surface varies with each injection, the LF signal provides immediate feedback throughout the assay steps. This capability ensures that the experiment is proceeding smoothly and allows for immediate quality control.

Figure 3a reports the sensograms of the complete assay for all the signal regions (Swt, So, and N curves) as well as for control regions (light green CTRL curves). All curves are presented with the standard error of the mean, indicated by lighter shading. All the designated steps are separated with the PBS 1 × washing.

The first step depicted corresponds to the blocking with BSA solution (c = 1 mg/mL). Although this step technically belongs to the bio-conjugation process, it is presented here to assess the reactivity of the signal regions of interest compared to the areas not bio-conjugated with protein solutions (control regions).

Subsequently, a positive serum sample (S) at a 1:10^3^ dilution is injected to interact with the immobilized protein sensing array. As can be seen, the regions exhibit different responses depending on the specific capture protein type.

After that, the biotinylated anti-human IgG (Bio-Rad Laboratories, Hercules, CA, USA, 1:20) is introduced. It produces a very small angular shift that is hardly quantified, proving that the LF mode is not sufficient for such a high dilution of human serum sample and that we need to switch to the FLR mode for a higher sensitivity. So, the FLR background is acquired (the first yellow band at t = 52 min). In order to remove any noise, this background signal is later subtracted from the signal obtained upon injection of the fluorescent labels.

Then, streptavidin-conjugated nanocrystals are introduced to react with the biotinylated anti-human IgG (QD step on the sensogram). These are QD655 from Life Technologies, Milano, Italy, selected to match spectral features of the FLR excitation/detection window of the read-out platform. Finally, a further fluorescence signal is acquired (the second yellow band).

By subtracting the BSA-reference (CTRL) sensograms from those of the signal regions (Swt, So, and N proteins), differential LF sensograms were obtained for the five signal regions, as shown in Figure 3b. The interaction with the positive serum sample (S) is evidenced by a pronounced differential shift associated with the N regions compared to the Swt and So regions (t = 26 min). The limited angular displacement observed for the So regions is consistent with previous findings [23], where, even at weaker (1:10^2^) dilution, the LF response was relatively weak. Interestingly, the N regions exhibit a strong signal (20 pix), reflecting the high concentration of anti-SARS-CoV-2 N antibodies typically present in the serum of infected patients. A further increase in the LF angular shift is observed following the injection of streptavidin-conjugated QDs. The N regions exhibit a net shift of ~30 pixels, while the Swt region shows a smaller shift of less than 10 pixels, and as expected, no appreciable shift is detected for the So regions.

However, as previously reported in [23], the detection limit of the LF mode for anti-SARS-CoV-2 S antibodies typically allows only a 1:10 serum dilution. Therefore, to quantify anti-SARS-CoV-2 S antibodies at higher dilutions, the FLR mode was employed, as it provides the ultra-sensitive detection capability required for highly diluted serum samples.

### 3.2. FLR-Based Detection

**Figure 4:**
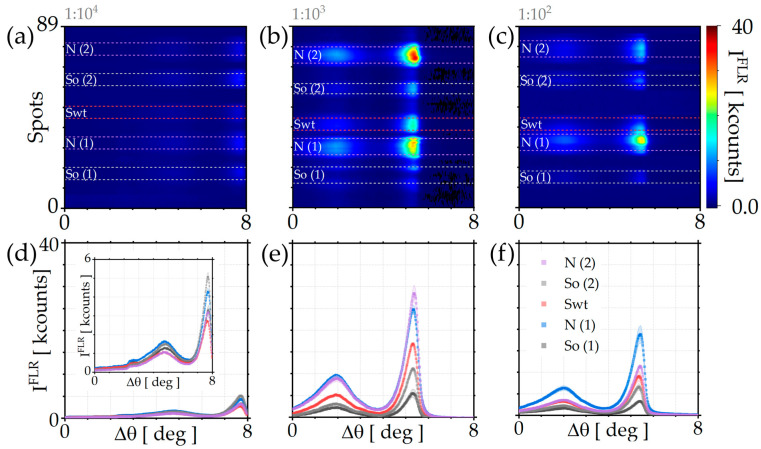
2D FLR maps obtained at serum dilutions of 1:10^4^ (**a**), 1:10^3^ (**b**), and 1:10^2^ (**c**). The signal regions are identified as N (1,2), So (1,2), and Swt, respectively. Subpanels (**d**–**f**) show the corresponding angular FLR spectra for the signal regions. The inset in subpanel (**d**) provides an enlarged view to better highlight the intensity of the TE peak associated with the 1:10^4^ FLR spectrum.

Fluorescence data are processed using a custom LabVIEW routine. Because the fluorescence response is angularly resolved, the biochip is scanned over multiple detection angles, and each angular profile is fitted to precisely extract the BSW resonance peak, corresponding to the maximum fluorescence coupling. Synthetic images are generated by mapping these peak intensities and applying system corrections, including subtraction of the pre-injection background and flat-field normalization. The resulting 2D FLR maps are shown in Figure 4a–c. For quantitative evaluation, the corrected fluorescence intensity from each of the longitudinal channel positions is integrated. The resulting profiles are fitted to identify the signal peaks. The final processed data are presented in Figure 4d–f.

In Figure 4a–c, the 2D FLR maps corresponding to serum dilutions of 1:10^4^, 1:10^3^, and 1:10^2^ are shown. The five signal regions are highlighted in different colors: Swt in red, N (1,2) in purple and blue, and So (1,2) in grey.

From Figure 4d–f, it can be observed, as expected, that the signal from the Swt region is higher than that from the So region. In the inset of Figure 4d, a magnified view of the plot is shown to better distinguish the FLR spectra corresponding to the different regions. This result reflects the higher prevalence of antibodies targeting the original spike protein (Swt), consistent with the fact that the infection wave preceded the global spread of the Omicron variant and the consequent emergence of variant-specific immune responses. As shown in Figure 4a–c, the 2D FLR maps at different serum dilutions reveal distinct fluorescence responses across the signal regions. To further quantify these differences, the TE peaks corresponding to the five signal regions are plotted as a function of serum dilution.

**Figure 5:**
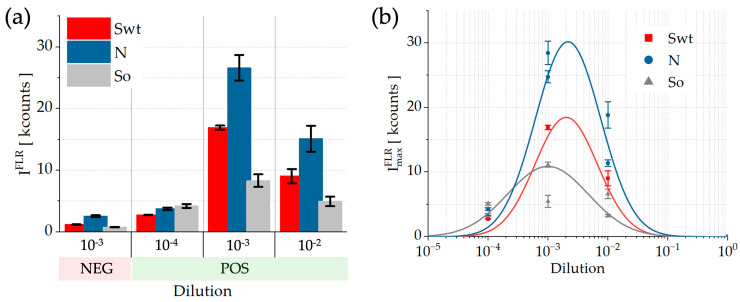
(**a**) Average TE peak intensities with standard deviations in the three regions (N, So, Swt) for positive serum dilutions (POS) 10^−4^–10^−2^ compared with a negative serum (NEG) dilution 10^−3^. (**b**) Maximum TE peak intensities for the five signal regions (N, Swt, and So). The fitting curves (log-normal functions) exhibit a maximum FLR intensity within a narrow dilution range centered around 10^–3^.

Figure 5a shows the average TE peak intensities in the three regions for the positive serum dilutions (POS), along with a histogram of the 1:10^3^ dilution for the negative serum sample (NEG), accompanied by their respective standard deviations, quantifying both spot-to-spot variability and the propagated uncertainty arising from the averaging of signals across equivalent regions on the same biochip. As evident, the positive serum signals consistently exceed those of the negative sample, demonstrating the capability of the platform to discriminate between positive and negative anti-SARS-CoV-2 IgG serum samples.

Figure 5b presents the maximum TE peak intensities ($I_{max}^{FLR}$) for the POS serum dilutions in the N, So, and Swt regions, as well complemented by their respective standard deviations. The analysis revealed an optimal dynamic range for FLR detection. From the observed logarithmic dependencies (log-normal fitting curve), it is possible to determine the dilution interval that yields the best fluorescence response for the N (blue), So (black) and Swt (red) regions. This behavior confirms that the FLR mode enables the specific detection of anti-SARS-CoV-2 antibodies across a well-defined range of serum dilutions (10^−4^–10^−2^).

From the intensity values obtained for the NEG and POS 10^−3^ dilutions, thus keeping the same sample complexity, it is possible to estimate the limit of detection (LoD), expressed in terms of dilution, for the three regions. The LoD extracted for Swt corresponds to approximately 0.7 × 10^−4^, slightly lower than the LoD values retrieved for the N and So regions, which are both around 0.95 × 10^−4^. The LoD values were determined by extrapolating the linear trends from the POS and NEG intensity points. These values are experimentally supported by the intensity measured for the POS 10^−4^ dilution, where the signal is clearly above the LoD, demonstrating the efficient detection of anti-SARS-CoV-2 antibodies at a 10^−4^ dilution. Overall, Figure 5b highlights an optimal dilution interval for fluorescence detection centered around 10^−3^, confirming this as the most effective working range for human-sera analysis.

This study makes it clear that a trade-off must be established between the detection of low-abundance biomarkers and the operational range of the FLR configuration, ensuring sufficient fluorescence collection from the 1DPC surface. Beyond their ability to distinguish positive and negative anti-SARS-CoV-2 antibodies in human serum, BSW-based biochips offer the significant advantage of inherent multiplexing capability. This feature paves the way for their use as diagnostic tools in detecting viral infections and for serological applications [21,30,31,32,33].

Moreover, the FLR mode supported by QD-based labels enhances photostability and promotes efficient energy transfer under resonant excitation, boosting emission even though the intrinsic excitation efficiency at λ_FLR_ = 637 nm is only 3%.

## 4. Conclusions

In summary, this study extends our previously established sensing platform by broadening its dynamic range for serological analysis. Through the implementation of the FLR mode, specific detection of anti-SARS-CoV-2 antibodies was achieved across a well-defined human serum concentration range (1:10^4^–1:10^2^). As demonstrated, the platform exhibits optimal performance at a serum dilution of 1:10^3^, allowing reliable discrimination among antibodies directed against the N, So, and Swt targets. Moreover, a clear and reliable discrimination between positive and negative human serum samples was achieved across all positive sample dilutions.

Furthermore, the inherent multiplexing capability of BSW-based biochips offers a key advantage, enabling their application in viral and serological diagnostics. Their low cost, high sensitivity, and reproducibility make them strong candidates for next-generation point-of-care testing.

## Data Availability

The data that support the findings of this study are available from the corresponding author, A.S., upon request.
